# The Promoter of the Oocyte-Specific Gene, *Oog1*, Functions in Both Male and Female Meiotic Germ Cells in Transgenic Mice

**DOI:** 10.1371/journal.pone.0068686

**Published:** 2013-07-22

**Authors:** Miya Ishida, Eriko Okazaki, Satoshi Tsukamoto, Koji Kimura, Akira Aizawa, Seiji Kito, Hiroshi Imai, Naojiro Minami

**Affiliations:** 1 Laboratory of Reproductive Biology, Graduate School of Agriculture, Kyoto University, Kyoto, Japan; 2 Laboratory of Animal and Genome Science Section, National Institute of Radiological Sciences, Chiba, Japan; 3 Animal Reproduction Laboratory, National Institute of Livestock and Grassland Science, National Agriculture and Food Research Organization, Nasushiobara, Japan; 4 Transposase JAPAN Inc., Showa, Gunma, Japan; Université Paris-Diderot, France

## Abstract

*Oog1* is an oocyte-specific gene whose expression is turned on in mouse oocytes at embryonic day (E) 15.5, concomitant with the time when most of the female germ cells stop proliferating and enter meiotic prophase. Here, we characterize the *Oog1* promoter, and show that transgenic *GFP* reporter expression driven by the 2.7 kb and 3.9 kb regions upstream of the *Oog1* transcription start site recapitulates the intrinsic *Oog1* expression pattern. In addition, the 3.9 kb upstream region exhibits stronger transcriptional activity than does the 2.7 kb region, suggesting that regulatory functions might be conserved in the additional 1.2 kb region found within the 3.9 kb promoter. Interestingly, the longer promoter (3.9 kb) also showed strong activity in male germ cells, from late pachytene spermatocytes to elongated spermatids. This is likely due to the aberrant demethylation of two CpG sites in the proximal promoter region. One was highly methylated in the tissues in which *GFP* expression was suppressed, and another was completely demethylated only in Oog1pro3.9 male and female germ cells. These results suggest that aberrant demethylation of the proximal promoter region induced ectopic expression in male germ cells under the control of 3.9 kb *Oog1* promoter. This is the first report indicating that sex-dependent gene expression is altered according to the length and the methylation status of the promoter region. Additionally, our results show that individual CpG sites are differentially methylated and play different roles in regulating promoter activity and gene transcription.

## Introduction

*Oogenesin1* (*Oog1*) is an oocyte-specific gene that is expressed after entry into meiosis and during early embryogenesis [1]. The mouse genome contains five copies of *Oog1* clustered on chromosomes 4 and 12. All of the copies contain a TATA-box in the proximal upstream region, suggesting that they are transcribed. *Oog1* expression begins in oocytes at E15.5 and continues to the 2-cell stage following fertilization [1]. Interestingly, OOG1 protein is localized in the nucleus of late 1-cell to early 2-cell embryos, concomitant with zygotic gene activation and first mitotic division. We previously identified a potential binding partner of OOG1, Ras and Ral guanine nucleotide dissociation stimulator (RalGDS), by yeast two-hybrid screening of a germinal vesicle (GV) oocyte cDNA library [2], but the function of *Oog1* remains unknown.

Understanding how oocyte-specific genes are transcriptionally controlled is important not only for uncovering mechanisms of oogenesis and early development, but also for generating useful tools to study gene function in oocytes. For instance, the *Gdf9* and *Zp3* promoters, which become active in mouse oocytes after birth, are frequently used for oocyte-specific transgenic and conditional KO studies [3–7]. Three other promoters (*H1oo*, *Npm2*, and *Zar1*) were recently shown by injection of luciferase reporter constructs into GV oocytes to drive reporter expression in oocytes [8]. Transgenic studies have shown that a relatively short core promoter region (~100 bp) is sufficient to induce germ cell–specific expression [9,10]. Germ cell–specific isoforms of transcription factors such as TRF2, TRF3, TAF4b, TAF7L, and ALF [11–13] likely bind to this core promoter region and induce germ cell–specific gene expression.

However, some transcription factors that bind proximal or distal regions outside of the core promoter strongly affect the expression of oocyte-specific genes [14,15], suggesting that the core promoter region might be insufficient to regulate spatiotemporal gene expression in oocytes. For example, FIGα is a beta helix-loop-helix transcription factor that binds to an E-box element (CANNTG) and regulates the coordinated expression of mouse zona pellucida genes [16]. FIGα deficiency causes downregulation of several oocyte-expressed genes, including *Mater, Dppa3/Stella*, and *Oct4* [17]. An E-box consensus sequence (CAGCTG) at -182 bp in the *Gdf9* promoter is also critical for inducing gene expression in oocytes [18]. Similarly, *Nobox*, an oocyte-specific homeobox transcription factor expressed as early as E15.5, has been shown by mutant analysis to be required for expression of many oocyte genes, including *Oog1*, in newborn mouse ovaries [14,19]. A NOBOX DNA binding element (NBE: 5´-TAATTG/A-3´) located at -1796 bp in the *Npm2* promoter is crucial for enhancing the basal transcriptional activity [8], indicating that distal regulatory regions are involved in oocyte-specific gene expression. In addition, the methylation status of promoter regions has been shown to control the sex-specific expression of germ cell–specific genes [20].

Here, we analyzed the promoter region of *Oog1*, by comparing the 5´-flanking sequences of the five *Oog1* copies in the mouse genome. We identified long conserved sequences with several gaps, two of which (2.7 kb and 3.9 kb in length) were used to drive expression of a *GFP* reporter gene in transgenic mice: transgenic mice with either the 2.7 kb (Oog1pro2.7) or 3.9 kb (Oog1pro3.9) *Oog1* upstream sequence. Both the 2.7 kb and 3.9 kb sequences showed functional promoter activity in mouse oocytes, possibly as early as E15.5, and will be useful for analyzing the function of genes expressed in oocytes. We also found that the 3.9 kb promoter functioned in male germ cells, and that the methylation status of the proximal promoter region differed between the Oog1pro2.7 and Oog1pro3.9 transgenes in male and female germ cells, suggesting that CpG methylation of the proximal region of the *Oog1* promoter may control gene expression in both male and female germ cells.

## Materials and Methods

### Generation of transgenic mice

*Oog1* 5'-flanking sequences (2688 bp and 3870 bp long) containing a TATA box on the 3'-end and restriction sites on both ends were amplified from mouse genomic DNA. The PCR products were purified, cleaved with restriction enzyme, and used to replace the CMV promoter in the pAcGFP1-mem vector plasmid (Clontech Laboratories, Mountain View, CA). Linearized transgene fragments were purified and microinjected into the male pronucleus of fertilized C57BL/6J mouse eggs (SLC japan, Shizuoka, Japan). Microinjected eggs were then transferred into oviducts of pseudopregnant ICR females (CLEA japan, Tokyo, Japan). Transgenic mice were identified by PCR genotyping using the following *AcGFP* primer pair: sense, 5´- CACATGAAGCAGCACGACTT -3´; antisense, 5´- TTGCCATCCTCCTTGAAATC -3´ (176 bp fragment). Four transgenic founders were obtained: one Oog1pro2.7 male, two Oog1pro3.9 males (lines A and C), and one Oog1pro3.9 female (line B). Transgenic female offspring obtained from crossing between transgenic founder animals and wild-type animals were used for studies. Since all three Oog1pro3.9 transgenic lines showed the same reporter expression in the ovary and testis, these lines were used interchangeably in our analyses.

### Cryosectioning

Tissue was fixed in cold 4% paraformaldehyde in PBS (pH 7.4) for 2 h. Ovaries were dehydrated in 20% sucrose in PBS overnight, and then 30% for 1–3 h until the tissue sank. Testes were fixed overnight in 4% paraformaldehyde in PBS containing 20% sucrose, and then were sunk in a 30% sucrose solution. Dehydrated samples were then placed in Tissue Tek O.C.T. compound (Sakura Finetechnical, Tokyo, Japan) and frozen for sectioning. Five µm thick sections on glass slides were placed in cold PBS in the dark and examined for GFP expression. After that, slides were mounted with Vectashield mounting medium with DAPI (Vector Laboratories, Burlingame, CA) and examined again for nuclear staining. GFP and DAPI signals were examined under a fluorescence microscope (BX50, OLYMPUS, Tokyo, Japan) equipped with appropriate filters (OLYMPUS filter sets U-MWIB and U-MWU).

### RNA isolation, cDNA synthesis, and reverse transcription polymerase chain reaction (RT-PCR)

Ovaries were obtained from different ages of transgenic mice; E15.5 fetus, newborn, 1-week-old, and 5-week-old female mice. Testis, liver, kidney, spleen, heart, lung, and brain were obtained from 5-week-old female and 10-week-old male mice. For oocytes samples, 3- to 5-week-old female mice were superovulated with intraperitoneal injections of 5 IU equine chorionic gonadotropin (eCG) (ASUKA Pharmaceutical, Tokyo, Japan). After 48 h from hormonal treatment, GV oocytes were recovered from ovaries. Oocytes were freed from the surrounding cumulus cells and used for RNA isolation. Total RNA was extracted with TRIzol (Invitrogen, Carlsbad, CA) from each tissue. After treatment with RNase-free DNase I (Roche, Indianapolis, IN), reverse transcription was performed on isolated RNA in a 20 µl reaction volume using ReverTra Ace (TOYOBO, Osaka, Japan) with random primers (Invitrogen). The following RT-PCR primers were used: *AcGFP1-mem*: sense, 5´- TGTTCACCGGCATCGTGCCC -3´; antisense, 5´- CTCGGCGCGCGACTTGTAGT -3´ (314 bp); *Oog1*, sense, 5´- AGGAGGCCTTCACTGATGGA -3´; antisense, 5´-GTCCTTCGCATGAAGGGCAG -3´ (346 bp); *β-actin* (internal control): sense, 5´- ATGAGCTGCGTGTGGCCCCT -3´; antisense, 5´- CGGAACCGCTCGTTGCCAAT -3´ (494 bp). For quantification, relative band intensities of PCR products were determined with a model 4.0 Atto densitograph (Atto, Tokyo, Japan). Intensities were normalized to the intensity of the *Actin* band, and the averages from two independent experiments were analyzed.

### Embryo culture

Four-to five-week-old female mice were superovulated with intraperitoneal injections of 5 IU eCG followed 48 h later by 5 IU human chorionic gonadotropin (hCG) (ASUKA Pharmaceutical). hCG injected mice were crossed with male mice, and 32 h after hCG injection, 2-cell embryos were flushed from the oviduct and cultured in modified KSOM medium. GFP fluorescence was observed after culture *in vitro* for 0 h (2-cell), 24 h (4-cell), 48 h (morula), and 72 h (blastocyst) using an inverted microscope (TMD300, Nikon, Tokyo, Japan) with a B2 filter set (Nikon).

### Bisulfite sequencing

Genomic DNA from transgenic testes and oocytes was processed with the MethylCode Bisulfite Conversion Kit (Invitrogen), according to the manufacturer’s instructions. Growing oocytes were collected from ovaries of 2-week-old female mice by dissection in HTF medium containing 0.1% hyaluronidase, 0.2% collagenase, and 0.25% DNase I. Ovulated MII oocytes were collected from ovaries of 4- to 5-week-old superovulated females 16 h after hCG injection. Converted DNA was amplified by hemi-nested PCR. The first round of PCR was performed using EpiTaq HS (Takara Bio Inc., Otsu, Japan) as follows: 30 cycles of 98 °C for 10 s, 55 °C for 30 s, and 72 °C for 120 s using 5´- AGGGTATATGAGGGAAATGAATTATAGG -3´ and 5´- TTTCAACCTATTTAATTCTTCTCATACAACACAAC -3´ (for the promoter region of the transgenes), 5´- ACTATAACTCCAAACTCCAAAAAACCTAAT -3´ (for intrinsic *Oog1* promoter), as primers. Then, a second round of PCR was performed using KOD -Plus- (TOYOBO, Japan), with the nested primer set of 5´- GAGAGTATTTGGGTGGAGTTTGTAG -3´ and 5´- ACCAAACAAATCAACTTAATTTCACC -3´. Amplified fragments were cloned into the pCR2.1-TOPO vector (Invitrogen) and sequenced. Sequence identity and methylation status of obtained sequences were analyzed using QUantification tool for Methylation Analysis (QUMA) (http://quma.cdb.riken.jp/).

### Statistical analyses

Differences in *GFP* mRNA expression levels between the transgenic mouse lines were analyzed using the Student’s t-test. Differences in the methylation status of each CpG or in the overall methylation status between Oog1pro2.7 and Oog1pro3.9 transgenic lines was analyzed statistically with the QUMA program, using the Fisher’s exact test for individual CpGs and the Mann–Whitney U test for overall methylation. For all analyses, the difference was considered significant when p<0.05.

### Ethical approval for the use of animals

All animal experiments were approved by the Animal Research Committee of Kyoto University (Permit Number: 24-17), and were performed in accordance with the committee’s guidelines.

## Results

### In silico analysis of the upstream sequences of *Oog1* gene

**Figure 1:**
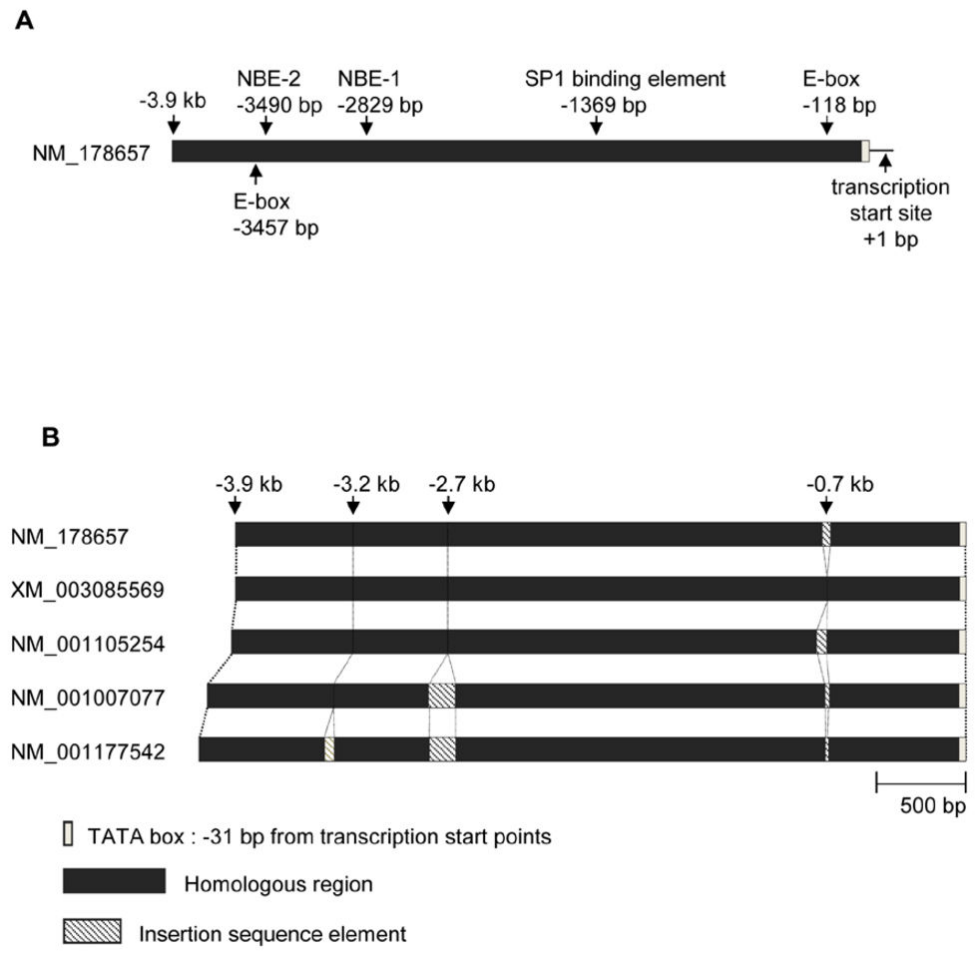
Schematic diagrams of the 5’-flanking sequences of the *Oog1* coding regions. A. Locations of putative transcription factor binding sites in the 3.9 kb *Oog1* promoter region (on chromosome 12, NT_039551) are shown by arrows. E-box (-188 bp), SP1 binding element (-1369 bp), and NBEs (-2829 bp and -3490 bp) are conserved among upstream regions of the five copies of *Oog1*. B. Promoter regions of the five copies of *Oog1*. All five sequences share a ~3.9 kb long, highly homologous region including a TATA box. Some sequences have sequence insertions at -0.7 kb, -2.7 kb, and -3.2 kb from the TATA box. The sequences on chromosome 4 (the upstream regions of NM_001007077 and NM_001177542) have the largest gaps at -2.7 kb.

*Oog1* is a multi-copy gene, with two copies on chromosome 4 [GenBank: NM_001007077, GenBank: NM_001177542] and three copies on chromosome 12 [GenBank: NM_178657 (*Oog1*), GenBank: XM_003085569, GenBank: NM_001105254]. Since all copies have a TATA box at -31 bp from the predicted transcription start site, they are likely all functional. Thus, we compared the upstream regions of all five copies of *Oog1* to identify the promoter region. Genomic sequence information for the 20 kb region upstream of each copy of *Oog1*, including the TATA box, was obtained from the NCBI (National Center for Biotechnology Information) database (http://www.ncbi.nlm.nih.gov/projects/mapview/). A homology comparison revealed that about 3.9 kb of the upstream sequence shared high homology between copies (Figure 1A). These sequences were then scanned for known transcription factor binding sites and annotated using the JASPAR CORE database (http://jaspar.genereg.net/). We found two Nobox binding sites (NOBOX DNA binding elements/NBEs; -2829 bp and -3490 bp) and one SP1 binding site (-1369 bp). In addition, we found eight E-boxes (or sequences similar to E-boxes) in the 3.9 kb promoter. However, only two of the E-boxes (-118 bp and -3457 bp from the transcriptional start site) retained perfect homology among the five sequences (Figure 1B). Furthermore, when the five promoters were compared with one another, inserted sequences at -0.7 kb, -2.7 kb, and -3.2 kb from the TATA box element were observed in some of the promoters (Figure 1A). The largest gap was seen -2.7 kb from the TATA box. Based on the above results, two candidate promoter sequences (2.7 kb and 3.9 kb in length) were isolated from the 5´-flanking sequence of *Oog1* (Gene ID: 193322; plus strand of chromosome 12) and were further analyzed for activity.

### The 2.7 kb and 3.9 kb promoter regions function specifically in oocytes of transgenic ovaries

**Figure 2:**
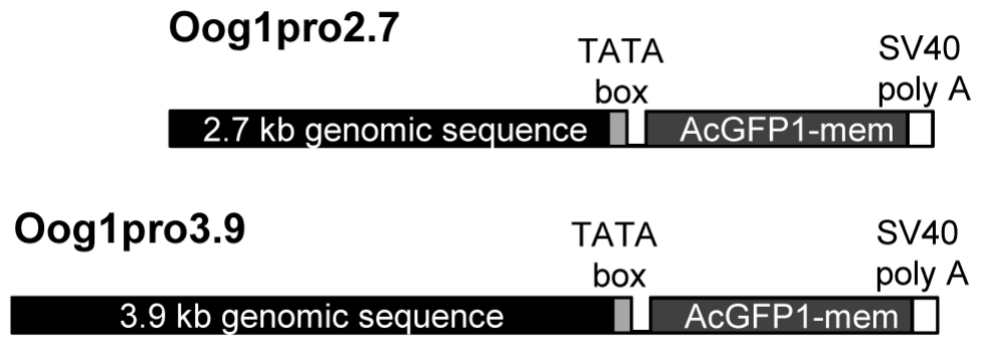
Transgene constructs for generating transgenic mice. Two constructs (Oog1pro2.7 and Oog1pro3.9) were used to generate transgenic mice.

**Figure 3:**
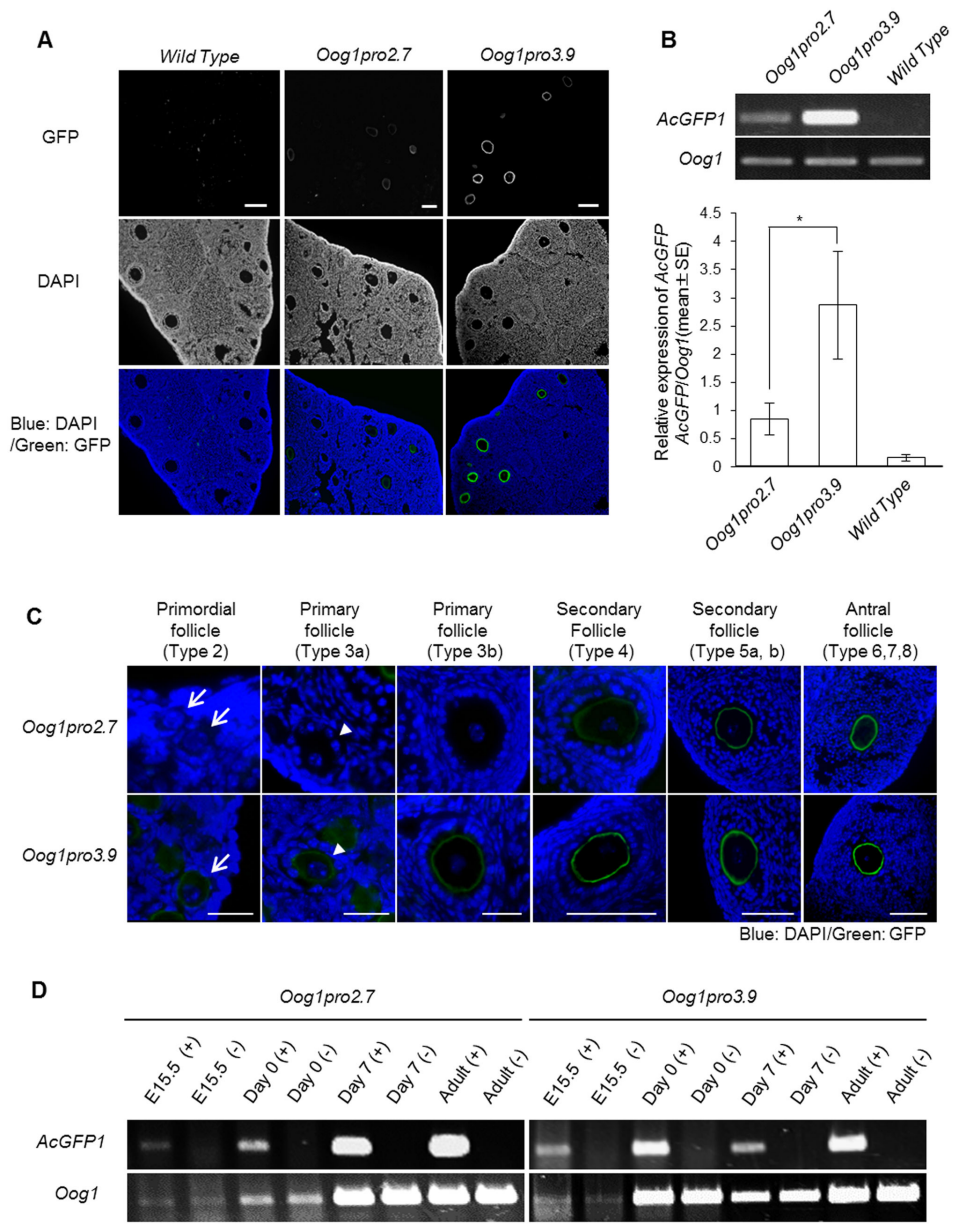
Oocyte-specific activities of *Oog1* promoters in transgenic ovaries. A. Frozen sections of ovaries obtained from 5-week-old transgenic mice. GFP signal was detected in the oocytes of both Oog1pro2.7 and Oog1pro3.9 transgenic mice. Because we employed membrane targeted GFP as the reporter gene, the green fluorescence signals were observed to concentrate around the plasma membrane of the oocytes. Scale bar: 100 µm. B. Quantification of *GFP* mRNA in 5-week ovaries of each transgenic line. The bar graph indicates the average value of the trials. RT-PCR was conducted twice using ovary cDNA obtained from two different animals per line, and similar results were obtained in each trial (* p<0.05, t-test). C. Magnified images of oocytes at various stages of folliculogenesis in transgenic ovaries. GFP signal was detected in the oocytes of secondary to preovulatory follicles in Oog1pro2.7 transgenic ovaries, but in the oocytes of primordial to preovulatory follicles in Oog1pro3.9 transgenic ovaries. Analysis of type 2 follicles was performed on sectioned 2-week old ovaries, and the remaining analyses were done using sectioned 5-week old ovaries. Arrows indicate primordial follicles; Arrowheads indicate primary follicles. Scale bars: primordial and primary follicles: 10 µm, secondary and antral follicles: 100 µm. D. RT-PCR analysis of transgenic ovaries using *AcGFP1-mem* and *Oog1* primers. Fetal (E15.5), neonatal (day 0), juvenile (day 7), and adult (5-week-old) ovary cDNA were obtained from transgenic (+) and non-transgenic (-) animals. *AcGFP1-mem* mRNA was detected in ovaries of all stages in Oog1pro2.7 and Oog1pro3.9 transgenic mice, similar to the pattern of *Oog1* mRNA expression.

We generated transgenic mice with either the 2.7 kb (Oog1pro2.7) or 3.9 kb (Oog1pro3.9) *Oog1* upstream sequence driving expression of a *GFP* reporter gene (Figure 2). Oocyte-specific GFP expression was observed in both Oog1pro2.7 and Oog1pro3.9 transgenic ovaries (Figure 3A). The intensity of the GFP signal in Oog1pro3.9 transgenic oocytes was stronger than that observed in Oog1pro2.7 transgenic oocytes. We confirmed by semi-quantitative RT-PCR that the promoter activity is about three times stronger in the Oog1pro3.9 ovary than in the Oog1prp2.7 ovary (Figure 3B). In addition, while GFP fluorescence in Oog1pro2.7 ovaries was observed in oocytes of type 4 secondary follicles and in subsequent stages of development, GFP signal was found in oocytes of type 2 primordial follicles in Oog1pro3.9 ovaries (Figure 3C). This difference may also be due to the difference in strength of transcriptional activity of each promoter.

Although these data suggest that the 2.7 kb and 3.9 kb sequences functioned specifically in oocytes within the ovary, GFP fluorescence was not observed in the oocytes within ovarian cysts (data not shown). Moreover, by Western blotting, GFP protein was not detected in newborn or fetal ovaries containing primordial follicles or ovarian cysts, respectively (data not shown). On the other hand, by RT-PCR, *GFP* transcripts were detected in E15.5 fetal transgenic ovaries, suggesting that both 2.7 kb and 3.9 kb promoters could function to produce mRNA in oocytes within the fetal ovary (Figure 3D). Indeed, the expression profiles of *GFP* mRNA in transgenic ovaries obtained at various stages from E15.5 to adult were similar to those of *Oog1*.

### The 2.7 kb and 3.9 kb promoters do not function in early embryos

**Figure 4:**
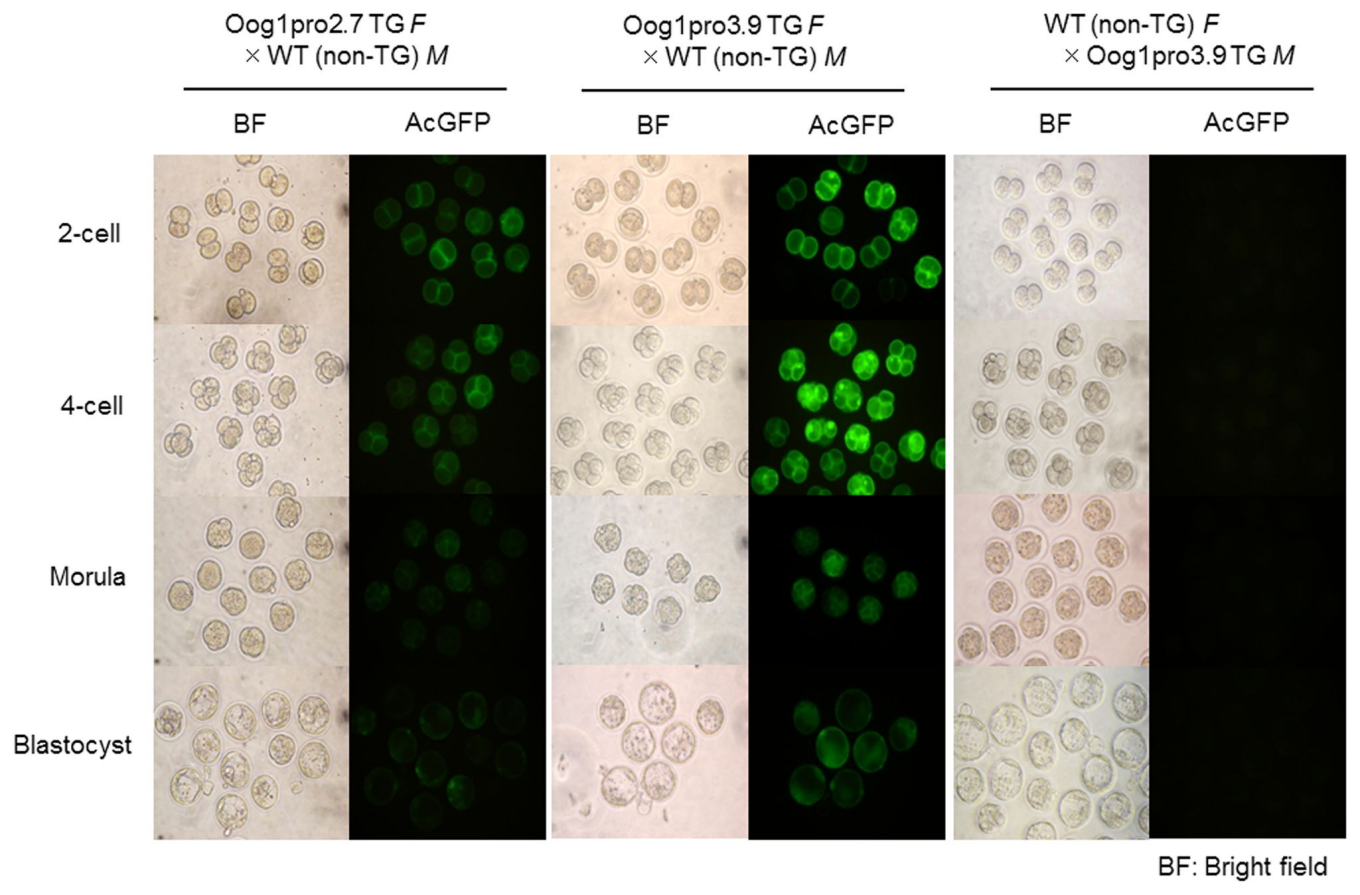
GFP signal in embryos derived from transgenic females. GFP signal was detected only in embryos obtained from transgenic females crossed with wild-type males. No signal was detected in embryos obtained from wild-type females crossed with transgenic males. Embryos were recovered at 1.5 days after hCG injection, and then were cultured for 3 days *in vitro*. Embryos at the 2-cell, 4-cell, morula, and blastocyst stages were observed at 1.5 days, 2.5 days, 3.5 days, and 4.5 days after hCG injection, respectively. *M*: Male, *F*: Female.

Since *Oog1* mRNA and protein are detected in early embryos until the late 2-cell stage [1], we examined the promoter activities during early preimplantation development (Figure 4). Appreciable fluorescence was observed in all zygotes derived from transgenic females crossed with wild-type males. The GFP signal decreased markedly at around morula stage, but was still detectable until the blastocyst stage. On the other hand, in embryos derived from non-transgenic females crossed with transgenic males, no GFP fluorescence was observed during preimplantation development. Because all transgenic males used for the experiments were confirmed to carry the transgenes and to successfully pass the transgenes on to their offspring, about half of the embryos collected in this experiment should carry the transgenic allele in their genome. In addition, the genes introduced into embryos by sperm will be activated at the time of zygotic gene activation (ZGA) and this occurs at the late 1-cell stage in the mouse. Thus, neither the 2.7 kb nor the 3.9 kb promoter appears to function after fertilization. These data suggest that *Oog1* transcript observed in early preimplantation embryos are of maternal origin.

### The longer promoter is active in male germ cells as well as in oocytes

**Figure 5:**
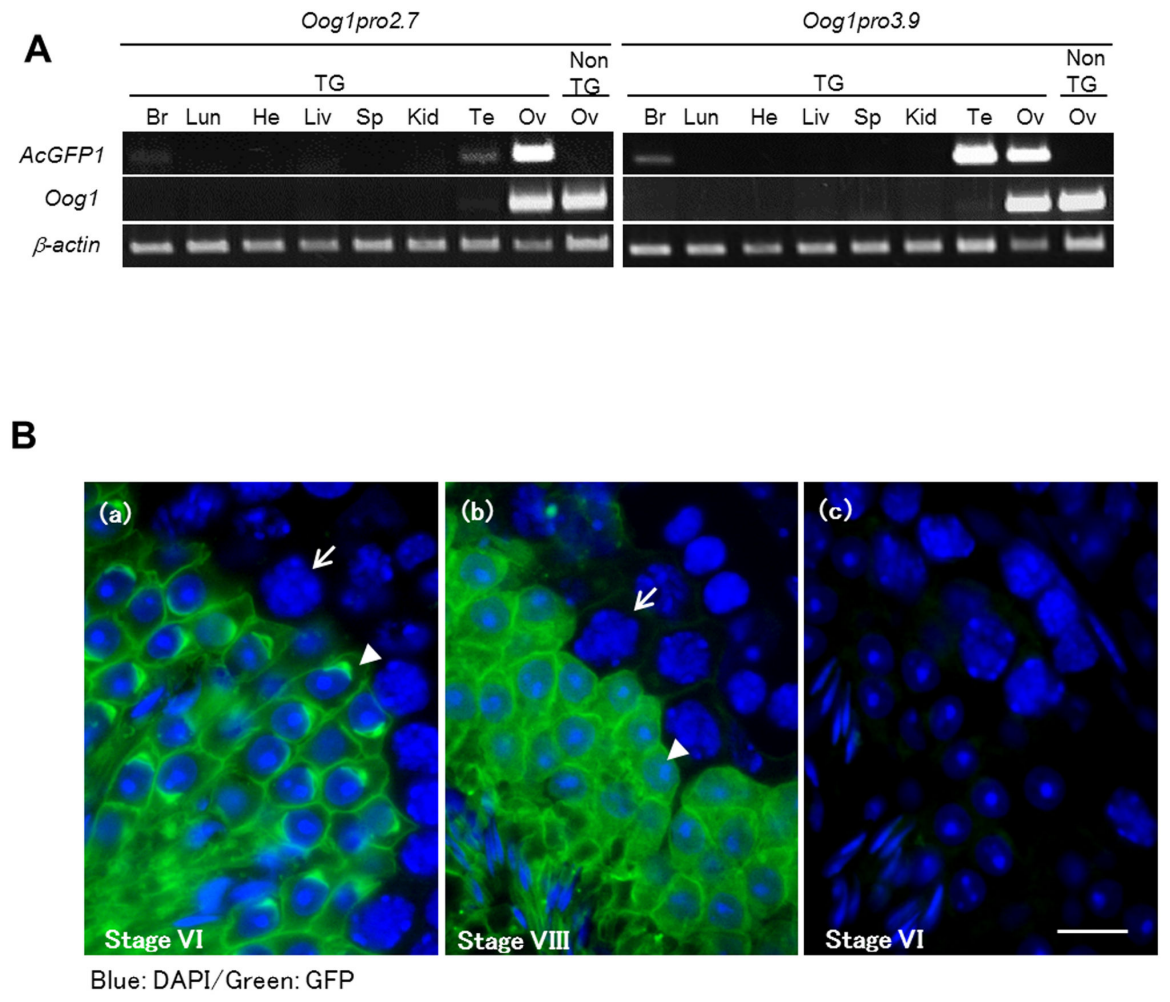
Activities of *Oog1* promoters in various tissues, including the testis. A. RT-PCR for *GFP* transcript in various somatic tissues of transgenic mice. Abundant *GFP* mRNA was detected in the ovaries of Oog1pro2.7 and Oog1pro3.9 transgenic mice, and in the testis of Oog1pro3.9 transgenic mice. Faint *GFP* mRNA expression was also detected in the brain in both transgenic lines, as well as in the testis of Oog1pro2.7 transgenic mice. Non-transgenic (NTG) ovary cDNA was used for controls. B. Frozen sections of the testis obtained from an Oog1pr3.9 transgenic male. (a) Seminiferous tubule at stage VI. GFP signal was detected in the round and elongated spermatids but not in mid-pachytene spermatocytes. Arrow: Mid pachytene spermatocyte; Arrow head: step 6 spermatid. (b) Seminiferous tubule at stage VIII. Late pachytene spermatocytes show the visible GFP signal. Arrow: Late pachytene spermatocyte; Arrow head: step 8 spermatid. (c) Seminiferous tubule of non-transgenic testis at stage VI is shown as a control. Scale bar: 10 µm.

Although the transcriptional activities of the 2.7 kb and 3.9 kb promoters differed, as shown by the transcript levels, both promoters functioned specifically in oocytes of ovarian cysts in transgenic ovaries. Unexpectedly, we also found by RT-PCR analysis of somatic tissues that the 3.9 kb promoter has strong transcriptional activity in the testis (Figure 5A). Whereas *GFP* mRNA was detected predominantly in the ovary in Oog1pro2.7 transgenic mice, it was detected in both female and male gonads in Oog1pro3.9 transgenic lines. GFP fluorescence was detected in male germ cells, from late pachytene spermatocytes to elongated spermatids, in Oog1pro3.9 transgenic testes (Figure 5B).

### Methylation status of the proximal region of the promoters affects sex-dependent gene expression

**Figure 6:**
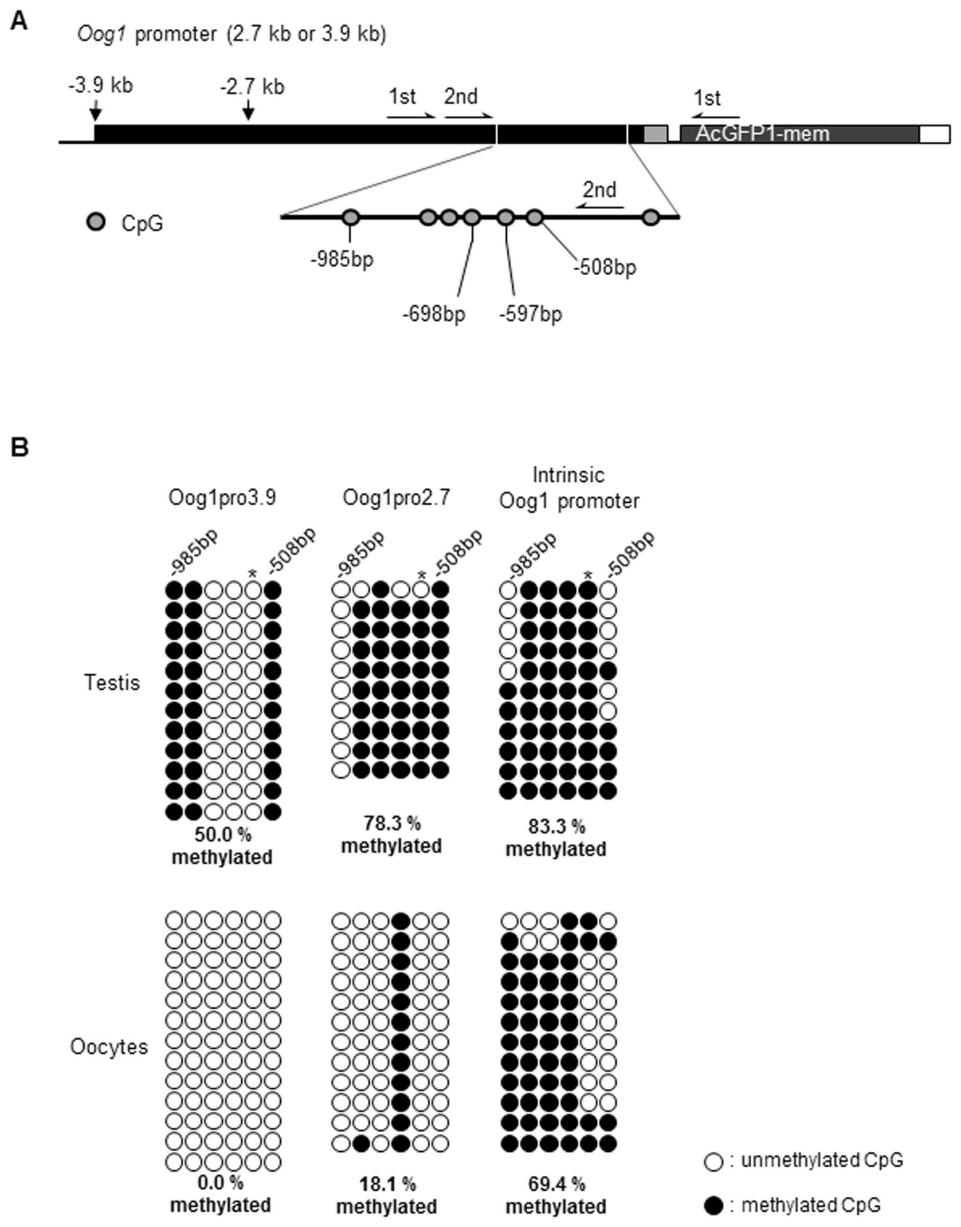
Bisulfite-sequencing analysis of the methylation status of the *Oog1* promoters. A. Location of CpGs and amplification primers on the *Oog1* promoter. The conserved 2.7 kb promoter region was analyzed with a program to predict DNA methylation (methylator, http://bio.dfci.harvard.edu/Methylator/index.html) and the indicated region (-508 bp to -985 bp) was selected for analysis of methylation status. Primers specifically recognizing the transgene were used for amplification of bisulfite-converted genomes (see materials and methods). B. DNA methylation status of individual CpGs is shown. Open circles indicate unmethylated, and filled circles indicate methylated CpG dinucleotides. Total methylation ratios were indicated under the diagrams. Asterisks indicate a significant difference in methylation status of individual CpGs between GFP expressed and non-expressed cells/tissues (p<0.05, Fisher’s exact test).

Since we observed differences in the tissue specificity of transgene expression between Oog1pro2.7 and Oog1pro3.9, we next analyzed the CpG methylation status of the promoter regions of Oog1pro2.7 and Oog1pro3.9. Bisulfite-sequencing of the proximal promoter region revealed that the cytosine of the CpG at -597 bp is highly methylated in tissues in which *GFP* expression was suppressed (Figure 6A, B), whereas the methylation ratio of the same cytosine was significantly reduced in tissues and cells in which *GFP* was expressed. Moreover, in both males and females, the cytosine of the CpG at -698 bp is highly methylated in the Oog1pro2.7 transgene and the endogenous Oog1 promoter, but is completely demethylated in the Oog1pro3.9 transgene, suggesting that the methylation of this cytosine is involved in repressing promoter activity only in male germ cells. Furthermore, the proximal promoter regions of the transgenes were highly methylated in somatic cells (Figure S1). These data suggest that the aberrant cytosine demethylation of two CpGs (at -587 bp and -698 bp) results in activation of the 3.9 kb promoter in Oog1pro3.9 male germ cells. Specifically, the cytosine methylation of a single CpG at -587 bp controls the basal promoter activity in both male and female germ cells, while cytosine methylation of the CpG at -698 bp is involved in suppressing aberrant expression in male germ cells.

## Discussion

Several germ cell–specific promoters have been isolated and used for the genetic analysis of germ cells [5]. So far, *Zp3* and *Gdf9* promoters are the only promoters known to have activity specifically in female germ cells, but they function only after birth [7]. Here, we isolated two *Oog1* promoter fragments (2.7 kb and 3.9 kb) and demonstrated that they function specifically in oocytes in the mouse ovary as early as E15.5. Our *in silico* analysis of the 3.9 kb promoter identified two E-box elements conserved perfectly among the five *Oog1* copies in the mouse genome. E-boxes are known to mediate differential gene expression by binding to homodimeric or heterodimeric complexes of beta helix-loop-helix transcription factors [21–24], such as FIGα [16], and play a key role in the regulation of oocyte-specific promoter activity [8,16,18,25]. Thus, the conserved E-box at -118 bp of both the 2.7 kb and 3.9 kb promoters may induce oocyte-specific transgenic expression.

We also found that Oog1pro3.9 drives stronger expression in oocytes than Oog1pro2.7. One possible explanation for this is that Oog1pro3.9 includes two NOBOX DNA binding elements (NBEs) (Figure 1A). Nobox is a homeobox transcription factor expressed in oocytes and can enhance the expression of oocyte-specific genes by binding to NBEs [8,14,19,26]. In *Nobox*-null newborn ovaries, the expression level of *Oog1* was dramatically reduced [19]. By contrast, there was little difference in *GFP* expression in GV oocytes between Oog1pro2.7 and Oog1pro3.9 (Figure S2). What might account for this discrepancy? During oogenesis, the rate of transcription decreases sharply when oocytes grow to their full size [27]. Concomitant with the decreased rate of transcription is a decrease in the concentrations of major transcription factors (TBP2 and SP1) during oocyte growth [13,28,29]. TBP2 (also known as TRF3) is preferentially expressed in germ cells in frogs and mice, and is replaced by TBP in growing oocytes [13,30]. SP1 binds to proximal binding sites, but can also interact with distal enhancer binding complexes to activate transcription, as shown for the IFN-β locus [31,32]. The fact that the *Oog1* promoter has a TATA-box and an SP1 binding site raises the possibility that enhancer complexes that bind to the 3.9 kb promoter function only during oocyte growth, and that this enhancer activity ceases in fully grown GV-stage oocytes, when the expression of TBP2 and SP1 decline significantly (Figure S3). This difference is also corroborated by the fact that the number of fully grown oocytes in the ovary is reduced by an order of magnitude as compared to that found in growing or non-growing oocytes [33].

Interestingly, Oog1pro3.9 also showed strong promoter activity in male germ cells during meiotic stages. *GFP* expression in Oog1pro3.9 males was detected in late pachytene spermatocytes (at stage VIII of spermatogenesis) and later on in elongated spermatids. Though this does not reflect the intrinsic *Oog1* expression pattern [1], it is intriguing that this ectopic expression in male germ cells is observed during meiotic stages. Since *Oog1* is normally expressed in female germ cells starting from stage E15.5, concomitant with the onset of the pachytene stage of meiotic prophase [34], this raises the possibility that *Oog1* plays a role in meiosis. However, this hypothesis needs to be tested directly.

The strong expression of Oog1pro3.9 in male germ cells cannot be explained by the presence of NBEs. Thus, there may be other regulatory functions in 1.2 kb sequence beyond the 2.7 kb promoter region, because 3.9 kb promoter drives germ cell–specific expression in both females and males. Thus, the expression of intrinsic *Oog1* might be controlled by multi-regulatory pathways, in which one pathway, such as the Nobox pathway, functions only in females, whereas the other functions in both sexes.

Methylation analysis of the Oog1pro2.7 and Oog1pro3.9 transgenes revealed that there is a significant difference in the methylation status of two CpGs (at -597 bp and -698 bp) in male and female germ cells. Thus, aberrant cytosine demethylation of these two CpGs in Oog1pro3.9 might cause *GFP* expression in male germ cells. A similar relationship between CpG methylation status and gene transcription was observed in the endogenous Oog1 promoter in the testis and oocytes. Promoter methylation and gene expression are known to be correlated [35–39], and tissue-specific differentially methylated regions (TDMs) are involved in the regulation of germ cell–specific gene expression [20,35]. Since the proximal promoter region of Oog1pro2.7 in testis has a similar methylation pattern to the endogenous *Oog1* promoter, it is possible that the regulatory elements in the distal promoter region in Oog1pro3.9 induced demethylation of the proximal promoter region, resulting in ectopic gene expression in the testis. The promoter region of Oog1pro3.9 has binding elements for two transcription factors, SP1 (5´-CCCCTTCCCC-3´) at -0.9 kb and NF-κB (5´-GGGAAATTCT-3´) at -3 kb. NF-κB can induce selective demethylation adjacent to its binding region [40] and can interact with SP1, which binds the proximal promoter region [41–43]. Because SP1 can mediate chromatin looping by interacting with distal enhancer complexes [31,32], it is possible that SP1 binds to the *Oog1* promoter and interacts with NF-κB as a trans-acting factor to demethylate the proximal promoter region, resulting in expression in Oog1pro3.9 male germ cells. Although the details remain to be investigated, our results suggest that CpG methylation of the proximal promoter region is involved in regulating the differential expression of *Oog1* in female germ cells.

Kido and Lau [44] showed that the promoter region that controls testis-specific protein Y-encoded (Tspy) gene can also function in female germ cells in transgenic mice expressing the *Cre* gene under the control of the *Tspy* promoter. In the case of *Tspy*, expression in females is avoided because the gene is located on the Y chromosome. In the case of *Oog1*, additional suppression mechanisms might be required to repress ectopic expression in male germ cells, since endogenous expression is restricted to female germ cells. Yan et al. reported that factors that interact with the *Gdf9* gene body are necessary to suppress *Gdf9* gene expression in male germ cells [18]. Thus, it is possible that *Oog1* expression in male germ cells is suppressed by factors interacting with the *Oog1* gene body*.*

We also confirmed that the 2.7 kb and 3.9 kb *Oog1* promoters do not function in preimplantation embryos. There was no GFP signal in embryos produced by crossing transgenic males with non-transgenic females. It has been reported that the expression of the gene derived from transgenic male is first detected at the late 1-cell stage in the mouse [45]. These results indicate that *Oog1* promoters are not activated after fertilization; rather, Oog1 protein observed in early embryos is likely translated from maternally inherited mRNA. Combined with its nuclear localization in the late 1-cell and early 2-cell stages, the possibility that *Oog1* plays a role in zygotic transcription at the 1- to 2-cell stage and/or in chromosome segregation of the first mitotic cell division as a maternal effect gene cannot be ruled out. Because oocyte-specific genes have been reported to be involved in oogenesis, fertilization, and early embryogenesis, characterizing the function of *Oog1* should help to elucidate the mechanisms of these biological phenomena *in vivo*.

Despite their importance, oocyte-expressed genes are difficult to study *in vitro* because there are no oocyte cell lines [9,18,46]. Although knockout (KO) and conditional KO animals are often used to analyze the functions of genes *in vivo*, this approach is not suitable for multi-copy genes such as *Oog1*. However, RNA interference (RNAi) constructs have been successfully used to study gene function in mouse oocytes [6,47,48] and could be effective for analyzing the function of multi-copy genes. Since this approach will likely require tight spatiotemporal control of RNAi constructs, the *Oog1* promoter will be very useful for future studies. We are currently generating transgenic mice using these newly identified promoters in order to knock down *Oog1* function during the first meiotic prophase in oocytes.

## Supporting Information

Figure S1 Bisulfite-sequencing analysis of the methylation status of the *Oog1* promoters in Oog1pro2.7 and Oog1pro3.9 livers. (TIF)

Figure S2 Semi-quantitative RT-PCR analysis of oocytes derived from transgenic mice. RT-PCR was conducted twice using different samples; each trial included 20 oocytes per sample. Similar results were obtained in each trial. The bar graph indicates the average value obtained from both trials. No significant differences were observed between Oog1pro2.7 and Oog1pro3.9 (n = 2, *t*-test).
(TIF)

Figure S3 A hypothetical model to explain the differences in promoter activities between Oog1pro2.7 and Oog1pro3.9 transgenic mice. (A) In growing oocytes, the oocyte-specific core transcription factor TBP2 is involved in maintaining basal transcription of the 2.7 kb and 3.9 kb *Oog1* promoters. SP1, which is also abundant in growing oocytes, interacts with the distal enhancer complex and upregulates transcription in the case of the 3.9 kb *Oog1* promoter. (B) In fully grown oocytes, the concentrations of TBP2 and SP1 proteins in oocytes are dramatically reduced, and the promoter activity is abrogated.
(TIF)
