# Supplementary figures and images for: The Promoter of the Oocyte-Specific Gene, Oog1, Functions in Both Male and Female Meiotic Germ Cells in Transgenic Mice
Source: PLoS One. 2013 Jul 22;8(7):e68686. doi: 10.1371/journal.pone.0068686 (PMC3718783; doi:10.1371/journal.pone.0068686)

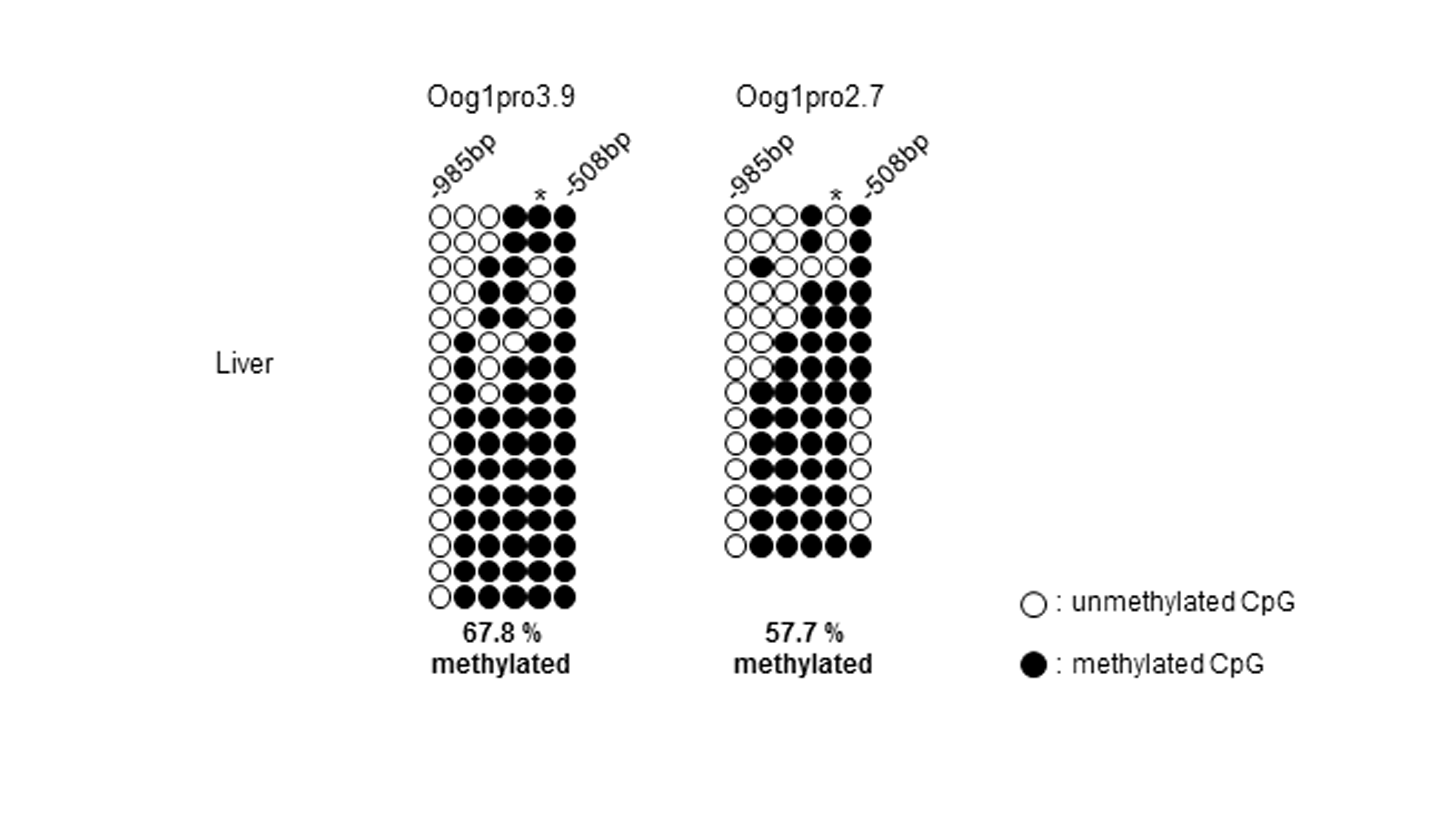

Supplement: Figure S1 — (TIF) [file pone.0068686.s001.tif]

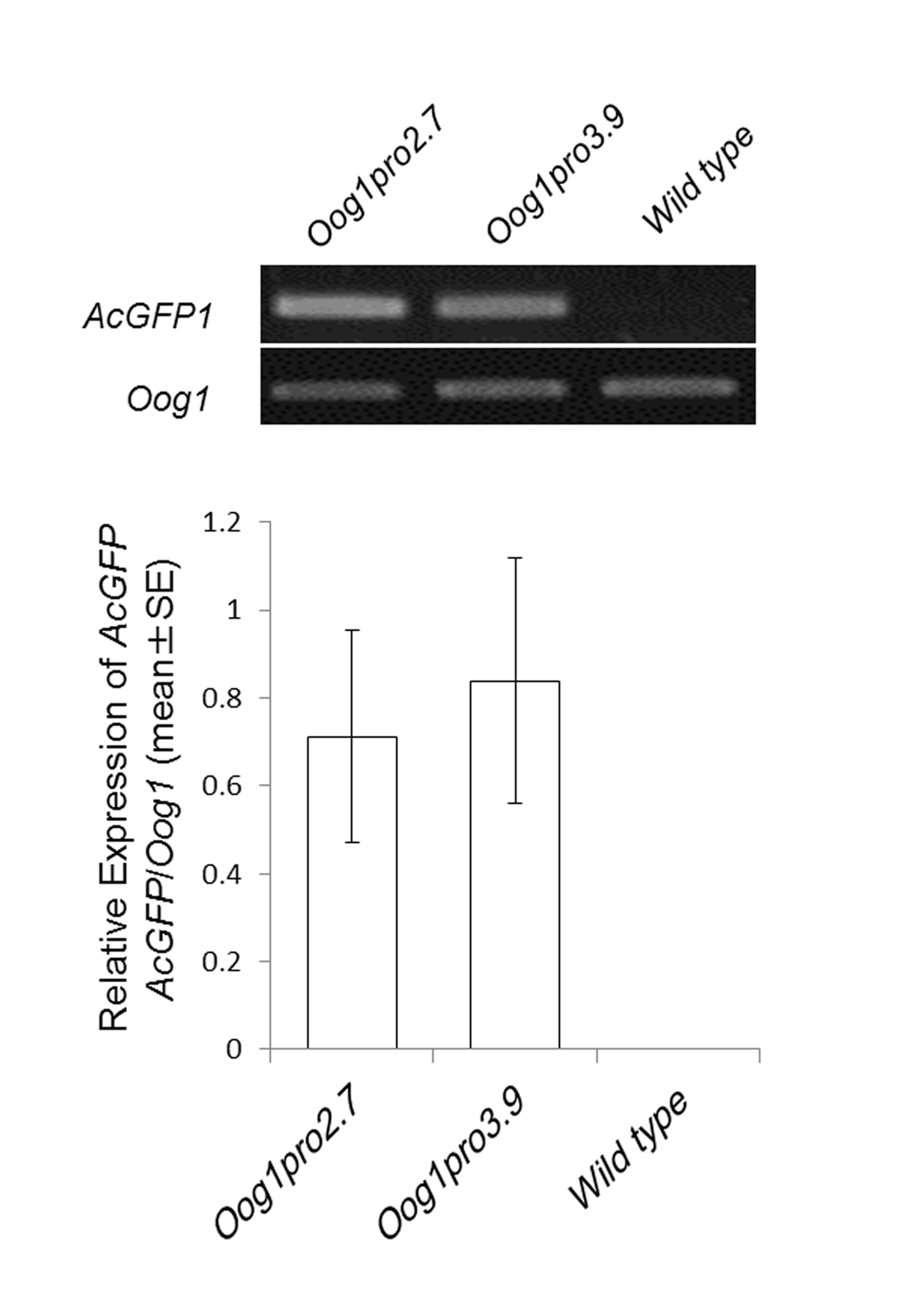

Supplement: Figure S2 — RT-PCR was conducted twice using different samples; each trial included 20 oocytes per sample. Similar results were obtained in each trial. The bar graph indicates the average value obtained from both trials. No significant differences were observed between Oog1pro2.7 and Oog1pro3.9 (n = 2, t-test). (TIF) [file pone.0068686.s002.tif]

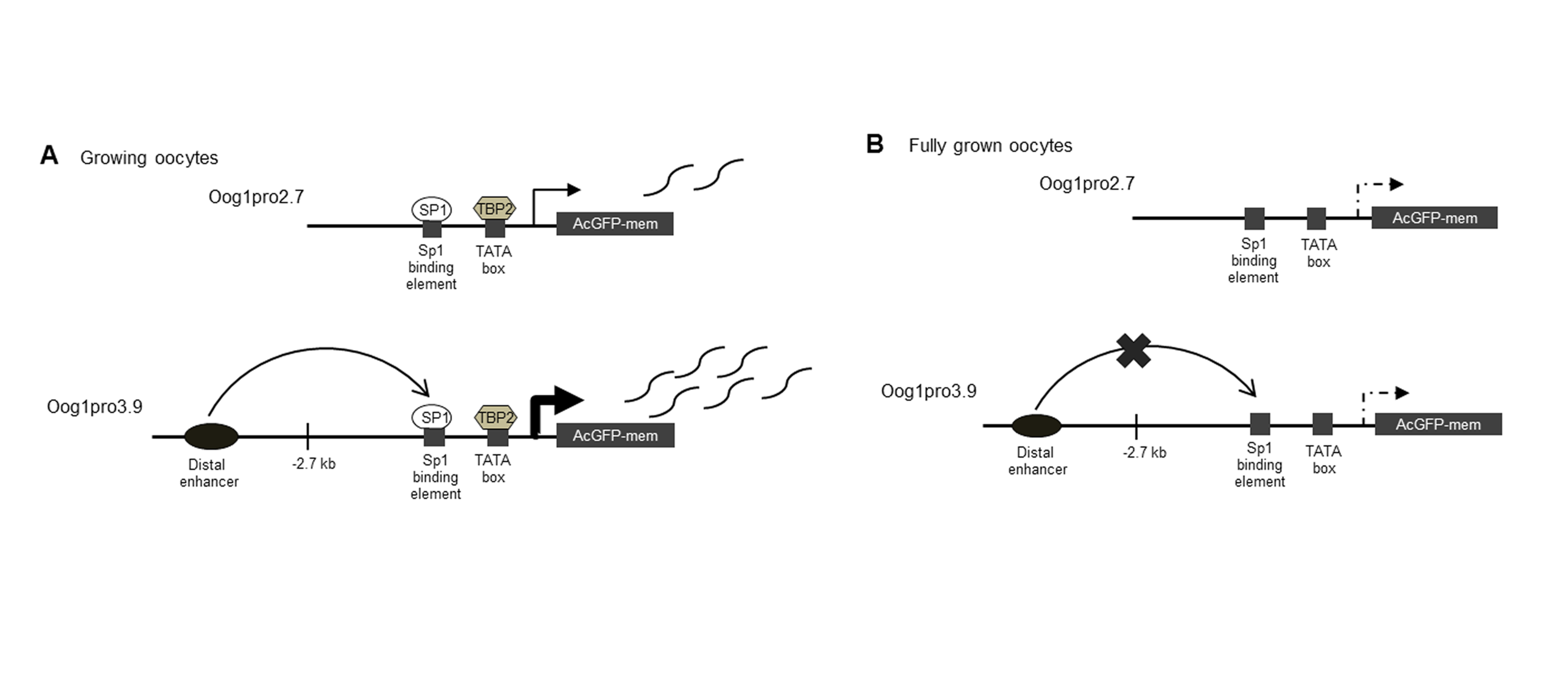

Supplement: Figure S3 — (A) In growing oocytes, the oocyte-specific core transcription factor TBP2 is involved in maintaining basal transcription of the 2.7 kb and 3.9 kb Oog1 promoters. SP1, which is also abundant in growing oocytes, interacts with the distal enhancer complex and upregulates transcription in the case of the 3.9 kb Oog1 promoter. (B) In fully grown oocytes, the concentrations of TBP2 and SP1 proteins in oocytes are dramatically reduced, and the promoter activity is abrogated. (TIF) [file pone.0068686.s003.tif]
